# Association between the atherogenic index of plasma and risk of large-artery atherosclerotic ischemic stroke

**DOI:** 10.3389/fneur.2025.1529628

**Published:** 2025-08-06

**Authors:** Wen Zhong, Nini Zhu, Xiaozhu Shen, Zhonglin Ge, Xiguang Liu, Guanghui Zhang, Qi Fang, Jingxian Liao

**Affiliations:** ^1^Department of Geriatrics, Lianyungang Hospital Affiliated to Jiangsu University, Lianyungang, China; ^2^Department of Geriatrics, Lianyungang Second People’s Hospital, Lianyungang, China; ^3^Department of Neurology, Lianyungang Second People’s Hospital, Lianyungang, China; ^4^Department of Neurosurgery, Lianyungang First People's Hospital, Lianyungang, China; ^5^Department of Neurology, The Affiliated Lianyungang Hospital of Xuzhou Medical University, Lianyungang, China; ^6^Department of Neurology, The First Affiliated Hospital of Soochow University, Suzhou, China

**Keywords:** atherogenic index of plasma (AIP), large artery atherosclerosis (LAA), atherosclerotic ischemic stroke (AIS), risk prediction, supplementary lipid metabolism

## Abstract

**Objective:**

Ischemic stroke caused by large artery atherosclerosis (LAA) is a major subtype of ischemic stroke and poses a heavy public health burden. Plasma atherogenic index (AIP) reflects the balance between pro- and anti-atherogenic lipid components and has emerged as a potential biomarker of cardiovascular disease. The aim of this study was to investigate the role of AIP in predicting ischemic stroke caused by LAA.

**Methods:**

This retrospective, cross-sectional study involved 2,382 ischemic stroke patients. AIP values were measured, and subjects were further stratified according to AIP levels. Univariate and multivariate logistic regression analyses were conducted to explore the relationship between AIP and the risk of LAA. Restricted cubic spline (RCS) analysis was used to detect the potential non-linear relationship, and receiver operating characteristic (ROC) curve analysis was performed to evaluate the predictive ability of AIP. Subgroup analyses were carried out to identify specific populations with a higher risk of LAA.

**Results:**

Individuals with consistently high levels of AIP were at increased risk of developing LAA, and this risk increased progressively with increasing levels of AIP. RCS analyses showed a threshold of 0.10 for the AIP index, a significant increase in the probability of LAA above this threshold, and a non-linear relationship between AIP and LAA. Univariate and multivariate logistic regression analyses showed that, as a continuous variable, each unit increase in AIP was significantly associated with an elevated risk of LAA. When divided into quartiles, the risk of LAA was higher in Q4 compared with the lowest quartile (Q1), and ROC curve analyses confirmed that AIP had moderate sensitivity and specificity in predicting LAA. Subgroup analyses showed that among individuals with consistently high AIP levels, those aged ≥60 years with a history of diabetes and low-density lipoprotein cholesterol (LDL-C) < 3.4 mmol/L were at higher risk of developing LAA.

**Conclusion:**

Herein, we found that elevated AIP levels are significantly associated with increased LAA risk and are an important biomarker to help identify patients at high risk for LAA.

## Introduction

Atherosclerotic ischemic stroke (AIS) remains a leading cause of morbidity and mortality worldwide, with its rising prevalence posing a significant public health challenge (1, 2). Atherosclerosis—the primary pathological basis of AIS—is characterized by lipid accumulation and chronic inflammation within arterial walls, resulting in plaque formation, luminal narrowing, and thromboembolic complications (3). Among various risk factors, dyslipidemia plays a pivotal role in the pathogenesis of AIS (4, 5). While reducing low-density lipoprotein cholesterol (LDL-C) has been associated with a decreased risk of ischemic events (6, 7), increasing evidence suggests that small dense LDL-C (sdLDL-C) possesses greater atherogenic potential (8).

Nevertheless, the complexity and cost-intensiveness of measuring sdLDL-C have limited its routine clinical use. Consequently, Burns et al. (9) proposed AIP, a novel lipid parameter calculated as the logarithm of the Triglycerides (TG)/High-density lipoprotein cholesterol (HDL-C) ratio, that can be easily determined using routine blood tests, as an alternative biomarker. In addition to reflecting sdLDL-C levels, AIP also evaluates cardiovascular risk. AIP has been recognized as a practical surrogate for sdLDL-C, effectively capturing the balance between atherogenic and anti-atherogenic lipid components (10–12). Elevated AIP has been associated with subclinical atherosclerosis, metabolic syndrome, and increased risk of cardiovascular and cerebrovascular diseases (13–15). Unlike conventional lipid indicators, AIP integrates two key lipid components and may offer superior predictive value in assessing atherosclerotic risk (16–18).

Despite growing interest, the predictive role of AIP in ischemic stroke remains underexplored—particularly in the context of large-artery atherosclerosis (LAA), the most common and debilitating AIS subtype. Most previous studies have evaluated general stroke populations without distinguishing between etiologic subtypes (19, 20), limiting the clinical applicability of AIP in risk stratification for specific stroke mechanisms.

To address this gap, the present study investigates the association between AIP levels and the occurrence of LAA-type AIS, as classified by the Trial of Org 10,172 in Acute Stroke Treatment (TOAST) criteria (21). A clearer understanding of this relationship may improve early identification of high-risk individuals and inform more targeted, lipid-focused strategies for stroke prevention.

## Materials and methods

### Study design and population

This retrospective study included adult patients diagnosed with AIS who were hospitalized in three tertiary medical centers in China: (1) the First Affiliated Hospital of Soochow University between March 2018 and January 2021, (2) the First People’s Hospital of Lianyungang between October 2019 and December 2021, and (3) the Second People’s Hospital of Lianyungang between January 2022 and July 2024. All participating hospitals used standardized diagnostic protocols and electronic health record systems to ensure data consistency. AIS diagnoses were confirmed by neurologists using clinical criteria in accordance with the World Health Organization definition and supported by neuroimaging (Computed Tomography or Magnetic Resonance Imaging). After admission, fasting blood samples were collected from all patients within 24 h by trained nursing staff for laboratory analysis, including lipid profiles and metabolic markers. Additional demographic and clinical data were extracted from hospital records by two independent investigators using a structured data collection form.

The inclusion criteria were: (1) Patients aged ≥ 18 years; (2) Patients who met the diagnostic criteria for AIS. On the other hand, the exclusion criteria were: (1) Patients with Intracranial Hemorrhage or mass bleeding; (2) Patients with severe infection or septic shock; (3) Patients with liver or kidney failure; (4) Patients with incomplete laboratory or clinical data; (5) Stroke of undetermined etiology.

This study was approved by the Ethics Committees of the First Affiliated Hospital of Soochow University (Approval No. 2020272, 2,019,057), the Second People’s Hospital of Lianyungang (Approval No. 2020050), and the First People’s Hospital of Lianyungang (Approval No. KY-20210917001-01).

### Baseline data collection

Experienced clinicians collected general patient information, including age, gender, Systolic Blood Pressure (SBP), Diastolic Blood Pressure (DBP), history of hypertension, history of diabetes, history of atrial fibrillation, smoking history, and alcohol consumption history. Within 24 h of admission, professional medical staff collected hematological samples for laboratory testing, including Total Cholesterol (TC), TG, LDL-C, HDL-C, Glucose (Glu), Glycated Hemoglobin (HbA1c), Albumin (Alb), Creatinine (Cr), and Homocysteine (Hcy).

Neurological status was assessed by two experienced neurologists using the National Institutes of Health Stroke Scale (NIHSS) at admission and discharge. Patients were categorized into large-artery atherosclerosis (LAA) or non-LAA subtypes based on the Trial of Org 10,172 in Acute Stroke Treatment (TOAST) criteria (21). Stroke severity was defined as mild (NIHSS < 4) or severe (NIHSS ≥ 4).

### AIP assessment

Herein, AIP values [log(TG/HDL-c)] were determined and grouped into quartiles (22): Q1:<−0.0904; Q2: −0.0904-0.0814; Q3:0.0814–0.255; and Q4: ≥ 0.255.

### Statistical analysis

All statistical analyses were conducted using R software (version 4.3.2). The Kolmogorov–Smirnov test was applied to assess the normality of continuous variables. Variables with normal distribution were summarized using mean ± standard deviation (SD), and comparisons between groups were made using independent samples *t*-tests. For non-normally distributed variables, data were presented as medians and interquartile ranges (IQRs), and group comparisons were performed using Mann–Whitney U tests. Categorical variables were expressed as counts (percentages), and intergroup differences were evaluated using the chi-square test or Fisher’s exact test when expected frequencies were <5.

Logistic regression analyses were used to examine the association between AIP and LAA, allowing adjustment for potential confounders. Multiple covariance tests were performed prior to the logistic regression analysis. Restricted cubic spline (RCS) analysis was conducted to explore the potential non-linear association between AIP and LAA risk. ROC curve analysis was performed to evaluate the discriminative power of AIP (23), with Youden’s index used to determine the optimal cutoff point.

Missing data were handled according to the percentage of incompleteness: variables with >10% missing data were excluded from analysis, while variables with <10% missing values were imputed using the Random Forest method via the mice package in R. All tests were two-tailed, and *p*-values <0.05 were considered statistically significant.

## Results

### Characteristics of study participants based on AIP quartiles

**Figure 1:**
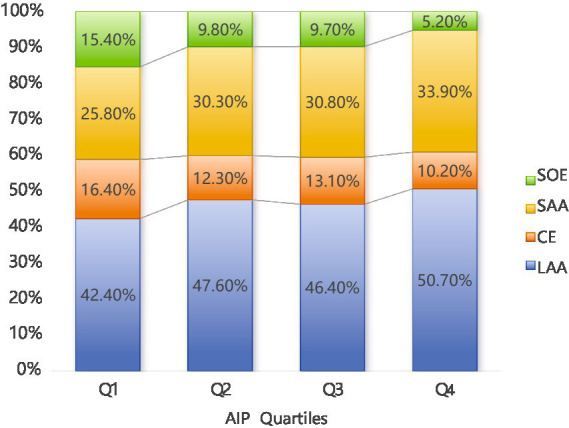
Percentage of AIP in the four quarters (Q1–Q4) according to Toast typing. LAA (Large Atherosclerotic Stroke) had the highest percentage in all quarters and amounted to 50.70% in Q4; LAA, large artery atherosclerotic; CE, cardiogenic; SAA, small artery occlusion; SOE, stroke of other etiology; AIP, atherogenic index of plasma.

Herein, as shown in Table 1, the baseline characteristics of participants were categorized by AIP quartiles, revealing significant differences across various demographic and clinical parameters. Among the total of 2,382 patients included in the study, 1,483 were male (62.3%) and 899 were female (37.7%). There was a notable trend in age distribution (*p* < 0.001), with median ages decreasing from Q1 (71 years) to Q4 (64 years). Furthermore, there was a decreasing trend in the proportion of participants aged ≥ 60 years across the quartiles, with Q1 and Q4 having the highest (84.2%) and lowest (63.8%) proportions, respectively. On the other hand, gender distribution was relatively balanced across the quartiles, although each group had more males, but with no significant intergroup difference (*p* = 0.085). Smoking and drinking habits increased considerably across the quartiles (*p* < 0.001 for both), with Q4 showing the highest proportions (47.5 and 56.7% for smoking and drinking, respectively). The prevalence of diabetes and atrial fibrillation also increased significantly (*p* < 0.001), with Q4 showing the highest prevalence (56.7 and 45.3%, respectively). On the other hand, the prevalence of hypertension remained consistent across groups (*p* = 0.119). Regarding metabolic markers, the quartiles showed significant differences in HbA1c, Alb, Glu, TC, TG, HDL-C, and LDL-C levels (all *p* < 0.001), with an upward trend in glucose and lipid indices from Q1 to Q4. Conversely, the quartiles showed no significant differences in SBP and Cr levels. Moreover, the median NIHSS scores at admission decreased slightly across quartiles (*p* = 0.008), with Q4 showing a larger proportion of mild cases (NIHSS < 4). As shown in Figure 1, according to the TOAST classification system, the groups exhibited an increase in LAA prevalence, with Q4 accounting for most cases (50.7%) (*p* = 0.040).

**Table 1:** Characteristics of study participants according to AIP quartiles.

Characteristic	AIP quartiles				*p*-value
	Q1	Q2	Q3	Q4	
Age, M (Q1, Q3) (years)	71 (65, 79)	70 (63, 77)	68 (59, 74)	64 (55, 71)	<0.001^***^
Age group, *n* (%)					<0.001^***^
≥60	502 (84.2%)	488 (82.0%)	437 (73.4%)	380 (63.8%)	
<60	94 (15.8%)	107(18.0%)	158 (26.6%)	216 (36.2%)	
Gender, male, *n* (%)	345 (57.9%)	377 (63.4%)	383 (64.4%)	378 (63.4%)	0.085
Smoking, *n* (%)	203 (34.1%)	231 (38.8%)	250 (42.0%)	304 (51.0%)	<0.001^***^
Drinking, *n* (%)	188 (31.5%)	201 (33.8%)	221 (37.1%)	283 (47.5%)	<0.001^***^
Diabetes, *n* (%)	207 (34.7%)	234 (39.3%)	264 (44.4%)	338 (56.7%)	<0.001^***^
Hypertension, *n* (%)	366 (61.4%)	378 (63.5%)	341 (57.3%)	349 (58.6%)	0.119
Atrial fibrillation	235 (39.4%)	225 (37.8%)	235 (39.5%)	270 (45.3%)	0.046^*^
SBP, M (Q1, Q3) (mmHg)	150 (137, 164)	149 (137, 162)	150 (137, 164)	150 (140, 163)	0.465
DBP, M (Q1, Q3) (mmHg)	85 (78, 96)	85 (78, 96)	87 (79, 96)	89 (80, 100)	0.059
HbA1c, M (Q1, Q3) (%)	5.90 (5.50, 6.30)	6.00 (5.60, 6.90)	6.30 (5.70, 7.60)	6.40 (5.70, 8.20)	<0.001^***^
Alb, M (Q1, Q3) (g/L)	38.4 (36.4, 40.2)	38.6 (37.0, 40.2)	39.3 (37.7, 40.8)	40.1 (38.6, 41.2)	<0.001^***^
Glu, M (Q1, Q3) (mmol/L)	5.46 (4.81, 6.87)	5.65 (5.01, 7.07)	5.91 (5.06, 7.75)	6.26 (5.22, 8.65)	<0.001^***^
TC, M (Q1, Q3) (mmol/L)	4.31 (3.61, 5.00)	4.28 (3.61, 4.94)	4.47 (3.80, 5.19)	4.73 (3.91, 5.45)	<0.001^***^
TG, M (Q1, Q3) (mmol/L)	0.77 (0.65, 0.91)	1.08 (0.96, 1.26)	1.48 (1.28, 1.71)	2.43 (2.00, 3.37)	<0.001^***^
HDL-C, M (Q1, Q3) (mmol/L)	1.30 (1.12, 1.49)	1.10 (0.99, 1.25)	1.02 (0.89, 1.15)	0.95 (0.81, 1.10)	<0.001^***^
LDL-C, M (Q1, Q3) (mmol/L)	2.47 (1.93, 3.05)	2.58 (2.06, 3.10)	2.75 (2.21, 3.24)	2.78 (2.27, 3.31)	<0.001^***^
Hcy, M (Q1, Q3) (μmol/L)	11.3 (8.9, 13.7)	11.2 (9.1, 14.0)	11.1 (9.0, 13.9)	10.4 (8.3, 13.6)	0.007^**^
Cr, M (Q1, Q3) (μmol/l)	85 (78, 96)	85 (78, 96)	87 (79, 96)	89 (80, 100)	0.657
NIHSS score at admission, M (Q1, Q3)	4.0 (2.0, 9.0)	3.0 (2.0, 8.0)	3.0 (1.0, 7.5)	3.0 (1.0, 7.0)	0.008^**^
NIHSS score at admission group, *n* (%)					0.107
<4	288 (48.3%)	306 (51.4%)	313 (52.6%)	330 (55.4%)	
≥4	308 (51.7%)	289 (48.6%)	282 (47.4%)	266 (44.6%)	
TOAST, *n* (%)					0.040^*^
LAA	253 (42.4%)	283 (47.6%)	276 (46.4%)	302 (50.7%)	
Non-LAA	343 (57.6%)	312 (52.4%)	319 (53.6%)	294 (49.3%)	

AIP, atherogenic index of plasma; SBP, systolic blood pressure; DBP, diastolic blood pressure; HbAlc, glycosylated hemoglobin; Alb, albumin; Glu, glucose; TC, cholesterol; TG, triglyceride; HDL-C, high density lipoprotein-cholesterol; LDL-C, low-density lipoprotein-cholesterol; Hcy, homocysteine; Cr, creatinine; TOAST, Trial of Org 10,172 in Acute Stroke Treatment; LAA, large artery atherosclerotic; NHISS, National Institute of Health stroke scale; *p < 0.05; **p < 0.01; ***p < 0.001.

### Univariate and multivariate logistic analyses of factors associated with LAA

**Figure 2:**
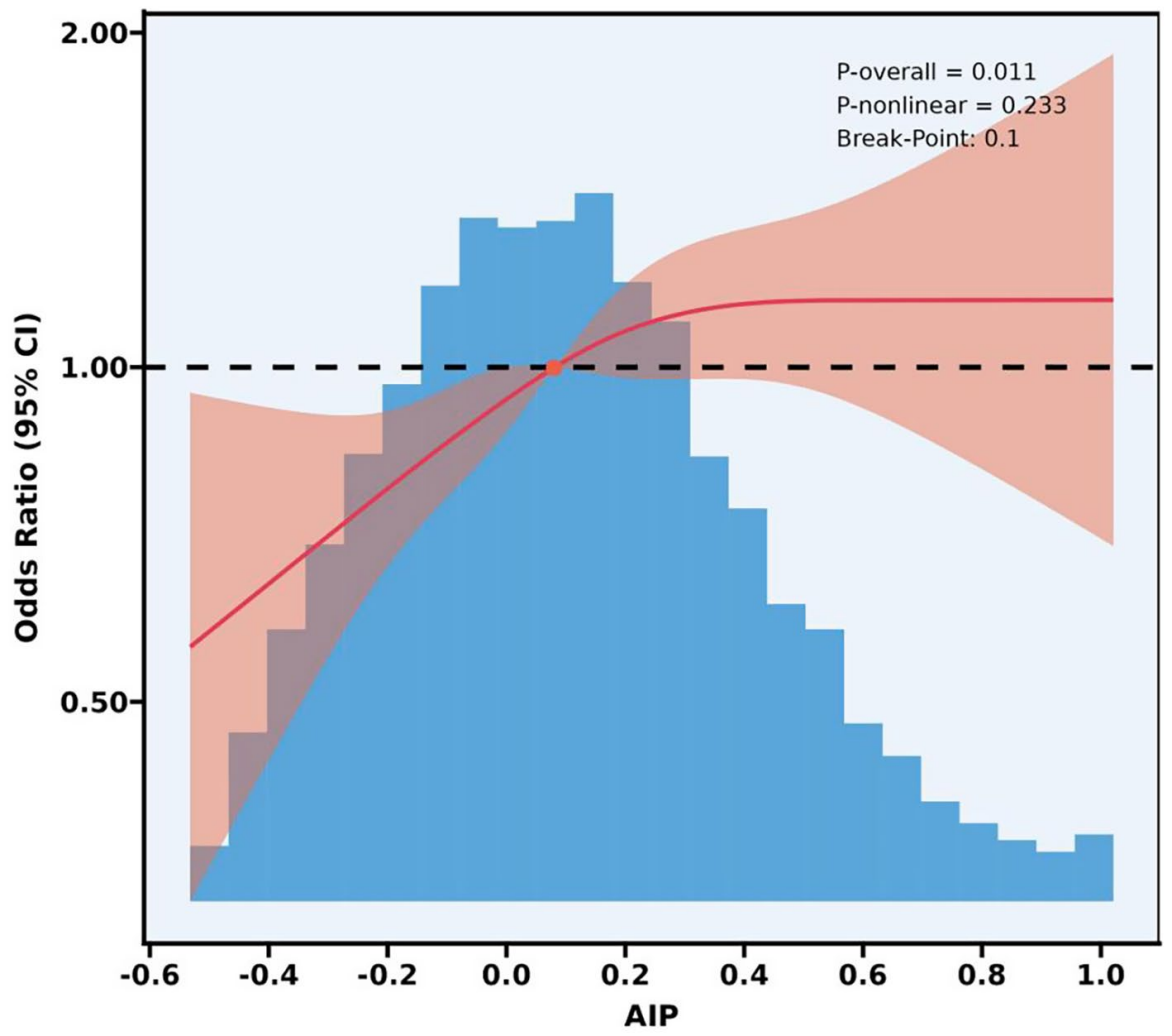
A restricted cubic spline analysis of the relationship between the AIP and LAA. AIP, atherogenic index of plasma; LAA, large artery atherosclerotic; CI, confidence interval.

Figure 2 shows a Restricted Cubic Spline (RCS) curve depicting the relationship between AIP and LAA. Notably, AIP correlated significantly positively with LAA (*p* = 0.011), with an inflection point observed at an AIP value of 0.10, after which the odds ratio (OR) increased significantly. Furthermore, the relationship between AIP and LAA did not deviate significantly from a linear trend (*p* for non-linearity = 0.233).

We assessed collinearity before analysis (Supplementary Table 1). We also assessed the association between various characteristics and LAA, as shown in Table 2, where NIHSS score at admission, smoking, HbAlc, Glu, TC, LDL-C, and AIP were independent risk factors for LAA. The risk of LAA occurrence increased by approximately 42% for each 1-unit increase in AIP when using AIP as a continuous variable [OR = 1.42, 95% confidence interval (CI): 1.07–1.89]. Categorical analysis of AIP showed that only Q4 was significantly associated with risk of LAA (OR = 1.39, 95% CI: 1.11–1.75).

**Table 2:** Univariate logistic regression analysis of the association between AIP and LAA.

Characteristic	OR	95%CI	*p*-value
Age	1.00	0.99, 1.01	0.726
NIHSS score at admission	1.04	1.02, 1.05	<0.001^***^
Smoking	1.19	1.01, 1.40	0.038^*^
Drinking	1.12	0.95, 1.32	0.192
Diabetes	0.99	0.85, 1.17	0.948
DBP	1.00	0.99, 1.01	0.944
HbAlc	1.09	1.04, 1.14	<0.001^***^
Alb	1.02	1.00, 1.04	0.067
Glu	1.05	1.02, 1.08	<0.001^***^
TC	1.15	1.07, 1.23	<0.001^***^
HDL-C	1.04	0.94, 1.14	0.462
LDL-C	1.32	1.20, 1.45	<0.001^***^
Hcy	1.00	0.99, 1.01	0.926
AIP (continuous)	1.42	1.07, 1.89	0.017^*^
AIP group			
Q1	-	-	
Q2	1.23	0.98, 1.55	0.076
Q3	1.17	0.93, 1.47	0.172
Q4	1.39	1.11, 1.75	0.004^**^

AIP, atherogenic index of plasma; DBP, diastolic blood pressure; HbAlc, glycosylated hemoglobin; Alb, albumin; Glu, glucose; TC, cholesterol; TG, triglyceride; HDL-C, high density lipoprotein-cholesterol; LDL-C, low-density lipoprotein-cholesterol; Hcy, homocysteine; NHISS, National Institute of Health stroke scale; OR, odds ratio; CI, confidence interval; *p < 0.05, **p < 0.01, ***p < 0.001.

This logistic regression analysis assessed the association between various characteristics and the likelihood of LAA, adjusting for different sets of covariates across five models, as shown in Table 3. When the AIP was treated as a continuous variable, each unit increase was associated with a higher risk of LAA, though the OR increased as more covariates were included: from OR = 1.42 in Model 1 to OR = 1.61 in Model 5. On the other hand, when analyzed categorically, Q4 of AIP showed a consistent and significant association with higher LAA odds across all models, with ORs ranging from 1.39 (95% CI:1.11–1.75, *p* = 0.004) in Model 1 to 1.32 (95% CI: 1.06–1.80, *p* = 0.005) in Model 5. Trend tests indicated a significant trend association between AIP groups and LAA across all models. These findings collectively suggest that higher AIP levels correlate with an increased risk of LAA.

**Table 3:** Multivariate logistic regression analysis of the association between AIP and LAA.

Characteristic	Model 1			Model 2			Model 3			Model 4			Model 5		
	OR	95% CI	*p*-value	OR^1^	95% CI	*p*-value	OR	95% CI	*p*-value	OR	95% CI	*p*-value	OR	95% CI	*p*-value
AIP (continuous)	1.42	1.07, 1.89	0.017^*^	1.43	1.07, 1.92	0.017^*^	1.43	1.06, 1.93	0.018^*^	1.48	1.09, 2.01	0.012^*^	1.61	1.02, 2.82	0.007^**^
AIP															
Q1	—	—		—	—		—	—		—	—		—	—	
Q2	1.23	0.98, 1.55	0.076	1.22	0.97,1.53	0.0.091	1.23	0.97, 1.55	0.081	1.2	0.95, 1.52	0.12	1.17	0.92, 1.49	0.211
Q3	1.17	0.93, 1.47	0.172	1.17	0.93,1.47	0.188	1.19	0.94, 1.50	0.149	1.18	0.93, 1.49	0.184	1.07	0.83, 1.38	0.618
Q4	1.39	1.11, 1.75	0.004^**^	1.4	1.11,1.77	0.005^**^	1.43	1.12, 1.81	0.004^**^	1.43	1.12, 1.82	0.004^**^	1.32	1.06, 1.80	0.005^**^
*P* for trend			0.010^*^			0.011^*^			0.007^**^			0.008^**^			0.013^*^

Model 1: no covariates were adjusted.

Model 2: adjusted for age and gender.

Model 3: adjusted for age, gender, NIHSS score at admission, SBP, DBP, smoking, and drinking.

Model 4: adjusted for age, gender, NIHSS score at admission, SBP, DBP, smoking, drinking, diabetes, hypertension, and atrial fibrillation.

Model 5: adjusted for age, gender, NIHSS score at admission, SBP, DBP, smoking, drinking, diabetes, hypertension, atrial fibrillation, HbAlc, Alb, Glu, TG, HDL, Hcy, and Cr.

OR, odds ratio; CI, confidence interval; *p < 0.05, **p < 0.01, ***p < 0.001.

### ROC analysis of the predictive value of AIP for LAA

**Figure 3:**
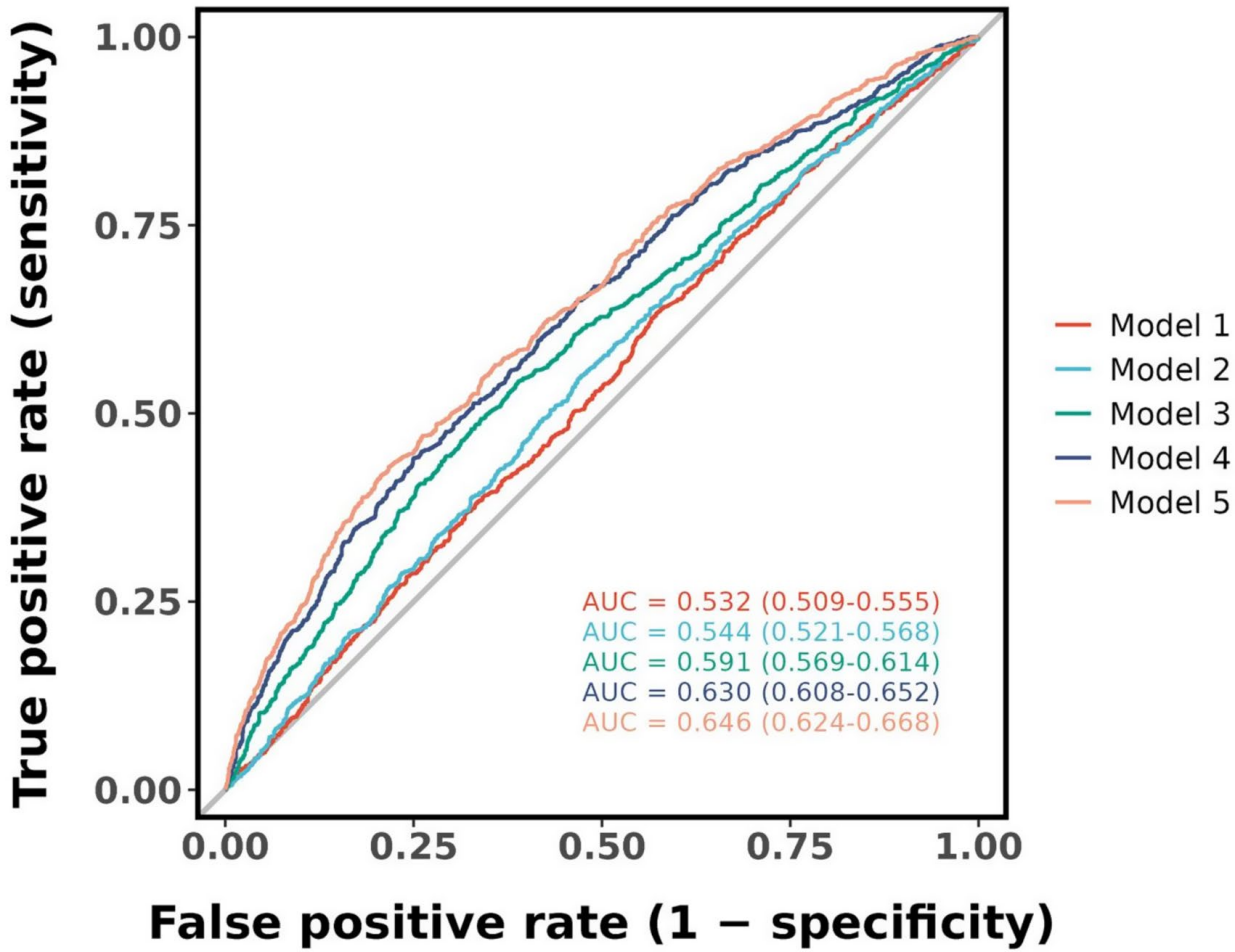
Receiver operating characteristic plot was generated depicting the predictive ability of the five models. AUC, area under curve.

Herein, an ROC plot was generated depicting the predictive ability of the five models in Figure 3. According to the plot, the area under curve (AUC) values increased gradually as covariates were progressively added from models 1 to 5, indicating an improvement in the models’ predictive value. The AUC value for Model 1 was 0.532, the optimal threshold value for the AIP index was 0.008, and the Yoden index, sensitivity and specificity values were 0.061, 63.9 and 42.2%, respectively. In Model 5, the AUC value was 0.646, the optimal threshold was 0.514, and the Youden’s index, sensitivity, and specificity values were 0.219, 45.5, and 76.4%, respectively.

### Subgroup analysis of LAA-related factors

According to the stratified analysis results, among individuals with consistently high AIP levels, the risk of developing LAA was higher in those aged ≥ 60 years compared to those aged < 60 years, and patients with diabetes had a higher risk of LAA than patients without diabetes, as well as in those with LDL-C < 3.4 mmol/L compared to those with LDL-C ≥ 3.4 mmol/L (both *p* < 0.05; Table 4). Furthermore, in the ≥ 60 years group, compared to Q1, the risk of LAA was higher in Q3 (OR = 1.31, 95% CI: 1.01–1.70) and Q4 (OR = 1.60, 95% CI: 1.22–2.09). In the LDL-C < 3.4 mmol/L group, only Q4 was significantly associated with LAA compared with Q1 (OR = 1.35, 95% CI: 1.05–1.74). On the other hand, in the LDL-C ≥ 3.4 mmol/L group, the risk of developing LAA in Q2, Q3, and Q4 did not change significantly relative to Q1 (all *p* > 0.05). In addition, in the group with an NIHSS score of ≥4 on admission, compared to Q1, compared with Q1, the risks of LAA in Q2, Q3, and Q4 increased by 34% (OR = 1.34, 95% CI: 1.01–1.85), 41% (OR = 1.41, 95% CI: 1.02–1.96), and 48% (OR = 1.48, 95% CI: 1.06–2.06). In contrast, only Q4 was associated with LAA in the NIHSS score at admission <4 group (OR = 1.41, 95% CI: 1.02–1.95).

**Table 4:** Subgroup analysis of LAA-related factors.

Subgroup	AIP quartiles				*p*-value
	Q1	Q2	Q3	Q4	
Age (years)					
<60	Reference	1.05(0.60–1.83)	0.82(0.49–1.38)	1.03(0.63–1.67)	0.454
≥60	Reference	1.27(0.99–1.63)	1.31(1.01–1.70)^*^	1.60(1.22–2.09)^***^	<0.001^***^
Diabetes					
Yes	Reference	1.44(0.98–2.09)	1.06(0.73–2.13)	1.50(1.06–2.13)^*^	0.022^*^
No	Reference	1.12(0.84–1.49)	1.29(0.96–1.73)	1.32(0.96–1.80)	0.088
LDL-C (mmol/L)					
<3.4	Reference	1.25(0.97–1.61)	1.20(0.93–1.55)	1.35(1.05–1.74)^*^	0.019^*^
≥3.4	Reference	0.97(0.55–1.73)	0.84(0.48–1.46)	1.24(0.71–2.15)	0.455
NIHSS score at admission					
<4	Reference	1.17(0.84–1.63)	1.02(0.74–1.43)	1.41(1.02–1.95)^*^	0.037^*^
≥4	Reference	1.34(1.01–1.85)^*^	1.41(1.02–1.96)^*^	1.48(1.06–2.06)^*^	0.020^*^

AIP, atherogenic index of plasma; LDL-C, low-density lipoprotein-cholesterol; NHISS, National Institute of Health stroke scale; *p < 0.05, **p < 0.01, ***p < 0.001.

## Discussion

This retrospective cross-sectional study identified a significant association between elevated AIP and increased risk of LAA in AIS patients, with a threshold effect at an AIP of 0.10. Univariate and multivariate logistic regression confirmed that each unit increase in AIP was associated with a 42% higher LAA risk (OR = 1.42, 95% CI: 1.07–1.89), with the highest quartile (Q4) showing a 39% increased risk (OR = 1.39, 95% CI: 1.11–1.75). These findings align with Zheng et al. prospective cohort study, where 6-year cumulative AIP exposure was linked to a 31% higher AIS risk, reinforcing AIP’s role as a key (but not exclusive) risk factor (19).

The AIP reflects the balance of pro-atherogenic triglycerides and protective HDL-C, driving formation of sdLDL-C particles that penetrate arterial walls and undergo oxidation (24). Concurrently, low HDL-C impairs reverse cholesterol transport, exacerbating lipid accumulation in atherosclerotic plaques (25). Mechanistically, AIP also correlates with oxidative stress and inflammation: elevated AIP triggers reactive oxygen species (ROS) production, damaging endothelial cells and promoting monocyte-derived macrophage activation—key steps in plaque instability (26, 27). Additionally, AIP-related lipid abnormalities may inhibit endothelial nitric oxide synthase (eNOS), reducing nitric oxide (NO) bioavailability—a critical factor in vasodilation and anti-thrombotic defense (28). These mechanisms collectively promote endothelial dysfunction, plaque instability, and heightened LAA risk.

Our subgroup analysis showed that among individuals with persistently high AIP levels, those with LDL-C < 3.4 mmol/L had a higher risk of developing LAA compared to those with LDL-C ≥ 3.4 mmol/L. This seemingly paradoxical result may be explained by the role of AIP in reflecting the formation of sdLDL-C particles, which are more atherogenic despite low total LDL-C levels (8, 29). Dobiásová and Frohlich (30) found that AIP is inversely correlated with LDL-C particle size and increases with the proportion of small-to-medium dense LDL-C. Thus, individuals with low LDL-C but elevated AIP may still carry a significant atherosclerotic burden due to sdLDL-C dominance. In contrast, in individuals with high LDL-C (≥3.4 mmol/L), the strong atherogenic potential of LDL-C may overshadow the added risk from AIP (30, 31). This is consistent with studies showing an inverse relationship between LDL particle size and atherosclerosis risk. While LDL-C are recognized risk factors for AIS (27), residual cardiovascular risk persists even when LDL-C is reduced to guideline-recommended levels (32–34).

The AIP, as a surrogate marker for sdLDL-C, offers a practical alternative. AIP correlates inversely with LDL-C particle size and could indirectly reflect sdLDL-C. Zhou et al. (35) found a positive correlation between sdLDL-C and IS risk, indicating that sdLDL-C could influence stroke progression and patient prognosis. Besides its long plasma half-life, SdLDL-C is resistant to hepatic clearance and prone to oxidation, potentially leading to foam cell formation. Furthermore, due to its small size, sdLDL-C is more likely to adhere to the vascular endothelium, leading to AS. It is also noteworthy that Wang et al. (36) reported a positive correlation between AIP and the risk of Early Neurological Deterioration (END) in AIS patients, with an optimal AIP threshold of 0.115 for predicting END. They also found that AIP had a higher predictive value for END than traditional lipid profiles (36). Additionally, several other studies have shown that AIP correlates with acute-phase neurological impairment and deterioration (37). Kokubo et al. (29) demonstrated that sdLDL-C is more predictive of cardiovascular events than total LDL-C or lipoprotein(a), though its routine measurement is limited by cost and technical complexity. As a cost-effective surrogate for sdLDL-C, AIP’s ease of measurement from routine blood tests supports its clinical utility.

In the ROC analysis, the Youden index was used to determine the optimal AIP threshold for identifying LAA. While the AIP cutoff in the unadjusted model (Model 1) demonstrated moderate sensitivity (63.9%), the specificity was relatively low (42.2%). With progressive covariate adjustment, specificity improved to 76.4% in Model 5, though sensitivity decreased to 45.5%. These findings indicate that although AIP may help identify individuals at higher risk of LAA, its limited specificity in certain models suggests it may be insufficient for use as an independent screening tool. Therefore, AIP may be more suitable as part of a multimodal risk assessment strategy that incorporates clinical features and other laboratory indicators to improve predictive accuracy.

Despite its strengths, this study has several limitations. First, as a cross-sectional study, it cannot establish causality between AIP and LAA. Second, some patients were on lipid-lowering medications, which may have influenced AIP levels and study outcomes. However, due to the retrospective nature of this study and the limitations of available medical records, detailed information regarding the type, dosage, and duration of lipid-lowering medications was not uniformly documented. As a result, potential confounding from lipid-lowering therapy could not be fully assessed. Third, the lack of follow-up precluded the evaluation of IS progression and long-term adverse outcomes. Future studies should incorporate longitudinal follow-up to assess the impact of AIP on stroke prognosis over time. Additionally, further research should explore the combined use of AIP with emerging biomarkers to improve stroke risk prediction and diagnostic accuracy. Investigating the impact of lifestyle modifications on AIP levels and subsequent stroke risk reduction could also provide valuable insights into preventive strategies.

## Conclusion

Our study demonstrates that elevated AIP levels are significantly associated with an increased risk of LAA, highlighting AIP as a crucial biomarker for early risk stratification. AIP reflects the balance between pro- and anti-atherogenic lipid components, with higher levels indicating a greater predisposition to atherosclerosis and stroke.

## Data Availability

The datasets used and/or analyzed during the current study are available from the corresponding author on reasonable request. Requests to access these datasets should be directed to Xiaozhu Shen, 499062796@qq.com.

## Glossary

Alb: Albumin
AIP: Atherogenic Index of Plasma
AIS: Atherosclerotic Ischemic Stroke
AUC: Area Under Curve
CE: Cardiogenic (TOAST subtype)
CI: Confidence Interval
Cr: Creatinine
cumAIP: Cumulative Exposure Index of AIP
DBP: Diastolic Blood Pressure
Glu: Glucose
HbA1c: Glycosylated Hemoglobin
HDL-C: High-Density Lipoprotein Cholesterol
Hcy: Homocysteine
IQRs: Interquartile Ranges
LDL-C: Low-Density Lipoprotein Cholesterol
LAA: Large Artery Atherosclerosis
NO: Nitric Oxide
NIHSS: National Institute of Health Stroke Scale
OS: Oxidative Stress
OR: Odds Ratio
RCS: Restricted Cubic Spline
ROS: Reactive Oxygen Species
ROC: Receiver Operating Characteristic
SAA: Small Artery Occlusion
SBP: Systolic Blood Pressure
SD: Standard Deviation
sdLDL-C: Small Dense Low-Density Lipoprotein Cholesterol
SOE: Stroke of Other Etiology
TC: Total Cholesterol
TG: Triglycerides
TOAST: Trial of Org 10,172 in Acute Stroke Treatment
eNOS: Endothelial Nitric Oxide Synthase
END: Early Neurological Deterioration
